# Epiploic steal: is this concept the key to reducing colorectal anastomotic leak rates?

**DOI:** 10.1007/s00423-024-03410-z

**Published:** 2024-07-13

**Authors:** Mina Sarofim

**Affiliations:** 1 Liver and Peritonectomy Unit, St George Hospital, Kogarah, NSW Australia; 2 School of Medicine, University of New South Wales, Sydney, Australia; 3 School of Medicine, The University of Sydney, Camperdown, NSW Australia

**Keywords:** Anastomotic leak, Anastomosis, Epiploic steal, Colorectal

## Abstract

**Purpose:**

Ensuring optimal colonic perfusion is a critical step in every colorectal anastomosis. The aim of this study is to describe the concept of epiploic steal.

**Methods:**

A literature review was performed to identify studies evaluating anastomotic blood supply. The fundamental principle of epiploic steal is outlined.

**Results:**

Epiploic steal has not been previously evaluated in the literature, and likely has a negative effect on colonic blood supply. Resection of colonic epiploicae may improve perfusion at the distal most lengths of a mobilised colonic conduit where the anastomosis requires it.

**Conclusion:**

This novel concept has the potential to change practice and reduce colorectal anastomotic leak rates. Further clinical studies are required.

The colorectal anastomosis has been described as the “apotheosis” of its specialty, with literature that suggests it is most successfully done in specialised hands. Anastomotic leaks have devastating septic sequela and are potentially lethal if not detected, in addition to increasing local recurrence when performed for neoplastic pathology [1].

Adequate perfusion is considered the fundamental step in anastomotic healing. The descending colon is supplied by the left colic artery, a branch of the inferior mesenteric artery (IMA). Additional supply is provided by the marginal artery and arc of Riolan which arise from the superior mesenteric artery. If high ligation of the IMA is performed, as commonly occurs in the setting of lymph node harvest in malignant disease, the distal descending colon relies on the additional supply rather than the natural left colic artery. Customary methods of assessing bowel viability (colour, bleeding, palpable pulses, or temperature) are subjective and not supported by a reliable evidence base [1, 2].

Tension, preoperative radiation, and intestinal microbiome also continue to play variably disputed roles in anastomotic leak rates. Handsewn or stapled suturing technique do not seem to bear significant impact [2, 3]. Acceptable leak rates for left-sided anastomoses range from 5 to 10%. Interestingly, despite numerous advances in bowel preparation, stapling devices, laparoscopy or robotic approaches, and perioperative management, the rates of anastomotic leak have not improved over the last 80 years.

Appendices epiploicae are a defining feature of the colon, which are peritoneal-lined serosal tags of adipose typically 2–5 cm long. In most anatomy textbooks they are relegated to a brief one or two-line description, largely due to their poorly understood function but they may act as mechanical cushions during peristalsis. They number approximately 50–100, are most abundant in the transverse, distal descending and sigmoid colon, and are supplied by branches of the circular artery of the corresponding segment of colon [4].

One may propose that precious blood flow of the mobilised left colon in a colorectal anastomosis should not be ‘wasted’ on perfusing dozens of epiploicae, but rather they should be resected such that flow is directed to the distal most lengths of the conduit where the anastomosis requires it. Extrapolated from the ‘vascular steal’ phenomenon, this hypothesis of epiploic steal has never been evaluated in colorectal surgery and thus never been proven, or disproven. A systematic review of the literature produces zero manuscripts on this topic.

Indocyanine green (ICG) fluorescence angiography is increasingly being used as an intraoperative adjunct to evaluate distal conduit perfusion in colorectal surgery as well as many other surgical specialties. A pilot study evaluating ICG perfusion of the gastric conduit during oesophagectomy reported that once the greater omentum was transected, conduit blood flow increased by 43–107% [5]. In a similar vein, removing unnecessary flow to the adipose of the colon may improve regional perfusion to optimise anastomotic healing.

Required is a randomised trial in colorectal patients which is certainly safe and feasible in combination with readily-available techniques such as ICG fluorescence: in one group conventional surgery, and in the other an energy device or ligatures used to resect the epiploicae along the conduit, foreseeably adding 5–10 min to operating time. However, the inevitable variables in chemo-radiation, surgical technique, tissue handling, and confounding patient atherosclerosis and haemodynamics – not to mention the large sample sizes required – would present significant challenges. Demonstrating a positive benefit is difficult when dealing with complications such as an anastomotic leak, which occurs as infrequently as 5%, but it may have a role in high risk patients.

Abolishing the risk of anastomotic leak is the enduring challenge in colorectal surgery, allowing us to offer patients safer and less morbid procedures, and lower rates of defunctioning stomas and their complications. As such, future evaluation of this novel epiploic steal hypothesis may represent the next core tenet of colorectal surgery.

## Data Availability

No datasets were generated or analysed during the current study.
